# Longitudinal standards for growth velocity of infants from birth to 4 years born in West Azerbaijan Province of northwest Iran

**DOI:** 10.4178/epih/e2015029

**Published:** 2015-06-23

**Authors:** Parvin Ghaemmaghami, Seyyed Mohammad Taghi Ayatollahi, Vahid Alinejad, Elham Haem

**Affiliations:** 1 Department of Biostatistics, Medical School, Shiraz University of Medical Sciences, Shiraz, Iran; 2 Patient Safety Research Center, Urmia University of Medical Sciences, Urmia, Iran

**Keywords:** Growth, Velocity, Weight, Length, Head circumference

## Abstract

**OBJECTIVES:**

Growth velocity is an important factor to monitor for appropriate child growth. This study presents the growth velocity of infants based on length, weight, and head circumference.

**METHODS:**

The subjects of this study were 308 neonates (160 boys and 148 girls) born in West Azerbaijan Province of northwestern Iran who were followed from birth for 4 years. The weights and lengths of the subjects were recorded at birth, 1, 2, 4, 6, and 9 months, and 1, 1.5, 2, 3, and 4 years of age, while the head circumferences were measured just up to 1.5 years of age. In this study, the Lambda-Mu-Sigma (LMS) method using LMS Chartmaker Pro (Institute of Child Health, London, UK) was utilized to obtain growth velocity percentiles.

**RESULTS:**

After obtaining growth velocity charts for weight, length, and head circumference (5th, 50th, and 95th percentiles), the researchers could deduce that there was a sharp decrease in the velocity growth charts from birth to 2 years of age but these charts remained relatively stable up to 4 years for both sexes. Growth velocities for the length and weight of boys in the present sample are slightly but not significantly greater than those in girls through the first months of infancy and there was no significant difference between girls and boys up to 4 years.

**CONCLUSIONS:**

This paper provided the first local growth velocity standards of length, weight, and head circumference for infants by analyzing longitudinal measurements produced for West Azerbaijan Province, which should be updated periodically. It seems that there has been a significant difference between the growth velocity of infants in northwestern Iran and southern Iran within the past few years.

## INTRODUCTION

Growth is one of the human body’s most complex processes. Each body part or region has its own unique growth patterns [1]. Monitoring health status, detection of growth failure, and determining the efficacy of interventions for a population can be performed by assessing the physical growth of infants and young children [2]. Monitoring physical growth and development is thus an important component of primary health care in pediatrics [3].

Anthropometric measurements are important indices of health in children and the most appropriate way to evaluate the nutritional and general health status of a community [1,3]. Weight, height, and head circumference are the most commonly measured parameters of infants’ physical growth [4].

Among the above-mentioned parameters, weight is the most widely used measurement since it is the simplest method of infant health assessment that is highly sensitive to short-term effects [4,5].

The next most-used measurement in the clinical environment is regular measurement of the head circumference, which is especially preferred among neonatologists and those caring for infants. The two groups of disorders evidenced by a large and small head can be detected by head circumference measurement [5,6]. Another worthwhile measurement is length because childhood stunting can manifest itself within the first two years of life, and it is through length measurement that changes in the velocity of growth can be detected and preventive measures can be applied [5].

Two valuable approaches to detecting changes in the growth of infants are a) comparison of the attained growth at a specific age with a reference chart, and b) growth velocity, which quantifies the growth changes within a time interval [7]. Velocity indicates the current status of the infant, whereas attained growth evidences the results of the infant’s entire life [4].

Attained growth is considered the most widely used means of evaluating whether a child’s growth is following a normal pattern or not. This approach analyzes growth with age in a static way. In contrast, growth velocity measurement is a dynamic process, which takes age into account over a period of time, and is more valuable in the assessment of growth compared with attained size [2,8,9].

Factors affect growth velocity directly while their effect on attained size only becomes apparent after altered rates of growth are constant for critical periods [5,10]. In other words, earlier recognition of growth problems are possible by assessing growth velocity rather than the sheer measurement of attained growth [5].

Due to lack of suitable longitudinal data sets, there are far fewer velocity references in comparison to attained growth. However, it would be time consuming to develop a reliable longitudinal growth standard because a large patient population, long follow-up times, and careful handling and analysis of data would then be needed [11].

In Iran, many studies have investigated the attained growth standards [6,12-15], but few surveys have investigated growth velocity standards [4,16]. To the best of our knowledge, at present, no study has assessed growth velocity in West Azerbaijan Province, northwest Iran. Therefore, this study aims to present growth velocity standards from longitudinally measured boys and girls of 0 to 4 years of age born in West Azerbaijan, for length, weight, and head circumference. We also compared the growth velocity for weight and length with our previously obtained charts in Shiraz, Fars, southern Iran [16], as well as published values from the UK [17].

## MATERIALS AND METHODS

The current study is longitudinal and includes measurements of weight, length, and head circumference of 308 healthy neonates (160 boys and 148 girls) who were randomly selected in a multistage sampling procedure from the nine counties (Urmia, Miandoab, Takab, Khoy, Mahabad, Bukan, Poldasht, Chaldoran, Salmas) of West Azerbaijan Province in 2008 and followed from birth until the age of 4 years at the health centers.

Among the 31 provinces in Iran, West Azerbaijan Province, is located in the northwest of Iran; it borders Turkey and has 27 counties. The province covers an area of 39,487 km², or 43,660 km² including Urmia Lake, with a population 3 million. Urmia is the capital and largest city of the province. Cold northern winds affect the province during winter and cause heavy snow [18].

The questionnaire that collects demographic information and neonates’ and their parents’ health status including sex, birth order, parents’ age, parents’ education, and anthropometric measurements was completed. Infants were visited at various target ages (birth, 1, 2, 4, 6, 9, 12, 18, 24, 36, and 48 months) and their weight, length, and head circumference were measured by trained health staff members. By using a baby scale until the second year of age, the weights were measured to the nearest 10 g and onwards to 0.1 kg. Using a supine position, the heights or lengths were measured until one year of age to the nearest 0.1 cm and then in a standing position, without shoes, in centimeters, using a SECA-marked stadiometer (SECA GmbH & Co. KG., Hamburg, Germany) and the techniques presented by Cameron, which have been fully described elsewhere [19]. The birth weights of 11 subjects (3.7%) were under 2,500 g (range, 1,300 g to 2,400 g).

Growth velocity was calculated as follows:

$$V=\frac{M_{n+1}\;-\;M_{n}}{\Delta t}$$

in which M_n_ and M_n+1_ were measurements at adjacent occasions, and *Δ*t was the time interval between them [20]. Because each measurement has its own measurement error, the variance of V is given by:

$$v\;(V)=\sigma^{2}+2\varepsilon^{2}/∆t$$

where *ε* is the measurement error and *σ* is the population standard deviation of the true measurement velocity. This indicates that the variability of velocity depends on the time interval, *Δt*, between measurements. The velocity standards must be constructed in a particular time interval. For anthropometric measurements during childhood, one year is common, which provides the advantage of removing any seasonal variation in measurement. It is worth mentioning that since one year is too long for length and weight measurement during infancy, it is suggested that intervals between 2 weeks and 3 months are more appropriate; however, infants are usually measured at irregular time intervals [20-22].

Then Lambda-Mu-Sigma (LMS) method was applied to calculate growth velocity curves for weight, length and head circumference measures, separately for each sex. The LMS method is a popular method to obtain smoothed percentile curves [23].

Each percentile curve is summarized by three curves representing the median (M), the coefficient of variation (S), and the skewness of distribution (L) as they change with the independent variable (age), the latter expressed as a Box-Cox power [17]. When the distribution is skewed and kurtotic, Z-scores do not have a valid interpretation. Thus we need to transform the distribution to (Gaussian or approximately so) before Z-scores can be correctly used. The LMS method applies here, primarily for correcting skewness. Using penalized likelihood, the three curves can be fitted as cubic splines by non-linear regression, and the extent of smoothing required can be expressed in terms of smoothing parameters or equivalent degrees of freedom [1].

The software packages used for construction of the growth velocity charts were the LMS Chartmaker Pro (Institute of Child Health, London, UK), SPSS version 11.5 (SPSS, Inc., Chicago, IL, USA), and Microsoft Office Excel (Microsoft Corp., Redmond, WA, USA).

## RESULTS

The number of subjects measured at various target ages are summarized in Table 1.

**Figure 1.**
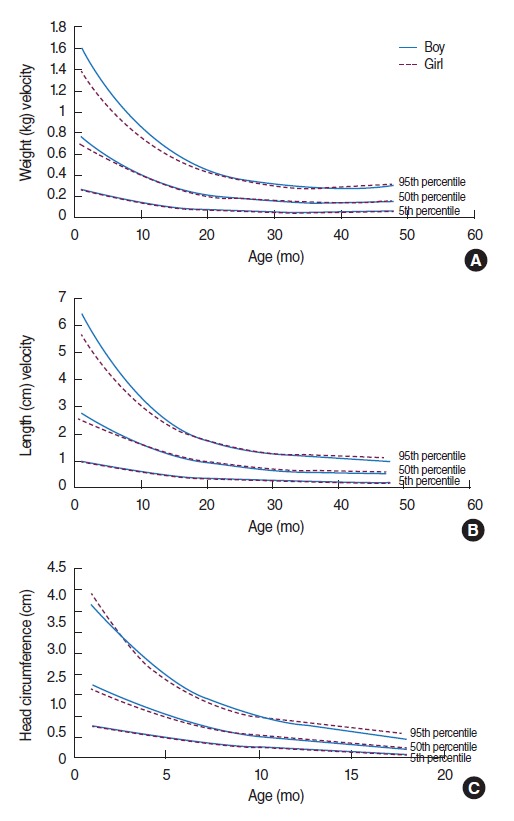
Velocity standards of infants in West Azerbaijan, Iran. (A) Weight (kg) velocity, (B) length (cm) velocity, and (C) head circumference (cm) velocity.

Figure 1A-1C shows velocity charts obtained for weight, length, and head circumference for median and extreme percentiles, respectively.

Figure1A and B illustrates the weight and length velocity charts of boys and girls. As can be seen, for all percentiles, boys’ charts and girls’ charts were similar, and in some parts the curves even overlay each other exactly, except for the younger children (less than 18 months), where the girls’ 95th percentile falls slightly below the boys’.

Figure 1C shows the head circumference growth velocity charts by sex. It shows that girls had higher velocity in the early months after birth. Then, they had a slightly lower velocity and after 12 months, girls had a higher velocity again in the 95th percentile. For the other percentiles, the boys’ and girls’ charts were close to each other, with the curves again overlaying each other exactly in some parts.

**Figure 2.**
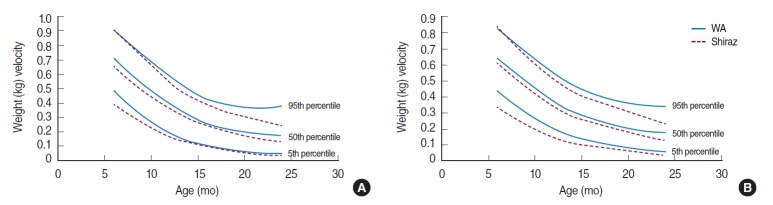
Weight (kg) velocity of infants in West Azerbaijan (WA) and Shiraz, Iran. (A) Males and (B) females.

**Figure 3.**
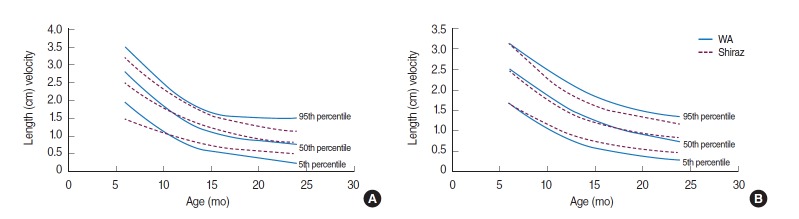
Length (cm) velocity of infants in West Azerbaijan (WA) and Shiraz, Iran. (A) Males and (B) females.

In addition, Figures 2 and 3 compare the velocity charts of weight and length in the present study with the charts of the Shiraz study carried out in 2005 [16].

Figure 2 shows that the growth velocity charts for weight in the south of Iran lie below those of northwest of Iran standards in both sexes.

## DISCUSSION

As screening tools, growth velocity standards are efficient indices to assess short-term changes in the growth rate of children, so they are usually taken into considering for primary health care in pediatrics. The present study used longitudinal data to estimate growth velocities from birth until the age of 4 years on a fairly large number of individuals.

Growth velocity measures were obtained during the last measured interval (1 month for the first 2 months of the infant’s life, bimonthly in the next 4 months, 3 months for the second 6 months, 6 months for the second year, and 12 months for the third and fourth years), whereas Shiraz data were available at different intervals [16].

Growth velocities for the length and weight of boys in the present sample are slightly but not significantly greater than those in girls through the first months of infancy, as can be seen in Figure 1A and 1B. Figures 2 and 3 compare weight and length velocity charts with the data from Shiraz for both sexes, respectively.

Weight velocity standards in the infants of this study were significantly higher than those in Shiraz, but similar to their UK counterparts and the differences were more pronounced in the first year of life [17]. Table 2 compares the median of length and weight velocities of West Azerbaijan, Shiraz, and UK infants by sex and age. These studies reported pronounced sex differences in the velocity measurements.

Our subjects were healthy infants without evidence of malnutrition who were monitored during the course of the study. The existing differences between the studied populations lead us to propose local growth velocity standards for the south and northwest of Iran separately.

The structural representativeness of our sample to that of the principal provinces of the northwest of Iran in terms of socioeconomic, demographic, and environmental factors suggest that our growth velocity standards are appropriate for the northwest of Iran. Therefore, the standards may not be representative of the entire population of Iran.

Growth of children in an urban region may differ from the growth of children who live in rural areas. Socio-environmental conditions as well as nutritional status may differ between children living in urban and rural areas [24,25]. An advantage of this study lies in providing a representative sample that comprises the majority of the regions (rural and urban) in a well designed longitudinal study, while the earlier surveys were based on infants visiting clinics in urban regions, who were unlikely to form a representative population sample. However, we suggest that due to changing characteristics over time, data from different parts of the country should be used. This way the local charts can be updated and even more representative.

Based on our findings, the growth velocities for the present sample are generally significantly greater than those in southern parts of Iran in both of two sex-age groups; this might be due to changes in lifestyle such as socioeconomic status, nutritional factors, living conditions, health and cultural developments due to modernization and rapid urbanization that have occurred in Iran in recent years. In addition, changing dietary habits, physical attributes, access to health care, and education can influence this secular trend in the growth velocity standard as a reliable measure for monitoring child growth [26,27]. Finally, this could be attributed to genetic, environmental, and infant feeding patterns. Therefore, our data supports the fact that an updated local standard for growth velocity should be used.

A major difference between the present study and previous ones is the statistical method that was applied for constructing growth velocity curves. In the present study, the parametric LMS method was used; many studies used this model to generate the growth velocity [7,28]. As Patel et al. [28] reported, the LMS model provided an accurate estimate of growth velocity. In the 2013 study of Ayatollahi et al. [29], the parametric LMS method was used to construct reference percentile curves for different measurements.

The LMS method has the advantage over other methods because after a suitable power transformation, the data are normally distributed; hence it delivers smoother percentiles. Furthermore, the three curves, L, M, and S, in the LMS method completely summarize the distribution of measurement over the wide range of covariates. In addition, since the LMS method assumes that after an appropriate power transformation, the data are normally distributed, anthropometric measurements, especially weight and height, tend to follow this pattern [30]. In a previous study [16], the non-parametric Healy-Rasbash-Yang method was used. However, before using this method on anthropometric measurements, some transformation such as a log transformation should be utilized for normalizing the variables.

Despite having major strengths with respect to collection of data in a relatively large cohort of neonates ages 0 to 4, this study also shows the inevitable obstacles and some limitations of such a long-term study. One limitation of our study is that, the nine counties are restricted to regions of West Azerbaijan; hence, it may not be representative of the entire population of children of West Azerbaijan Province. Another limitation is that, we did not account for the effect of important confounding factors such as body weight and body mass index; hence, we recommended using more reliable and accurate methods than just considering the age of children in future studies.

In conclusion, this paper provided the first local growth velocity standards of length, weight, and head circumference for infants by analyzing longitudinal measurements produced for West Azerbaijan Province (northwest Iran), which should be updated periodically. It seems that there has been a significant difference between the growth velocity of infants in the northwest of Iran compared with those in the southern area within the past few years. However, further research is needed on Iranian children in different provinces to determine the most reliable and valid index representing accurate assessment of growth velocity.

## Figures and Tables

**Table 1.** Number of subjects measured at various target ages

	Age										
	At birth	1 mo	2 mo	4 mo	6 mo	9 mo	12 mo	18 mo	24 mo	36 mo	48 mo
Males	160	155	156	155	152	151	149	150	147	145	139
Females	148	145	141	139	140	137	136	135	132	124	117
Total	308	300	297	294	292	288	285	285	279	269	256
Missing (%)	_	2.6	3.6	4.5	5.2	6.5	7.5	7.5	9.4	12.7	16.9

**Table 2.** Comparison of 50th percentile (median) of length and weight velocities of West Azerbaijan (WA) Shiraz, and UK infants, by sex and age

	Length velocity (cm/yr)						Weight velocity (kg/yr)					
	Males			Females			Males			Females		
	WA	Shiraz	UK	WA	Shiraz	UK	WA	Shiraz	UK	WA	Shiraz	UK
1st year	26.0	24.4	24.2	24.0	23.8	23.0	6.7	6.1	6.7	6.1	5.7	6.3
2nd year	11.0	11.7	10.6	11.5	11.2	11.4	2.5	2.3	2.5	2.5	2.2	2.5
